# Transfection of Platyhelminthes

**DOI:** 10.1155/2015/206161

**Published:** 2015-05-18

**Authors:** Bárbara Moguel, Raúl J. Bobes, Julio C. Carrero, Juan P. Laclette

**Affiliations:** Department of Immunology, Institute for Biomedical Research, National Autonomous University of Mexico, 04510 Mexico, DF, Mexico

## Abstract

Flatworms are one of the most diverse groups within Lophotrochozoa with more than 20,000 known species, distributed worldwide in different ecosystems, from the free-living organisms in the seas and lakes to highly specialized parasites living in a variety of hosts, including humans. Several infections caused by flatworms are considered major neglected diseases affecting countries in the Americas, Asia, and Africa. For several decades, a particular interest on free-living flatworms was due to their ability to regenerate considerable portions of the body, implying the presence of germ cells that could be important for medicine. The relevance of reverse genetics for this group is clear; understanding the phenotypic characteristics of specific genes will shed light on developmental traits of free-living and parasite worms. The genetic manipulation of flatworms will allow learning more about the mechanisms for tissue regeneration, designing new and more effective anthelmintic drugs, and explaining the host-parasite molecular crosstalk so far partially inaccessible for experimentation. In this review, availability of transfection techniques is analyzed across flatworms, from the initial transient achievements to the stable manipulations now developed for free-living and parasite species.

## 1. Platyhelminth Transfection Studies

The phylum Platyhelminthes or flatworms represent one of the most diverse groups within Lophotrochozoa with about 20,000 species distributed worldwide including free-living and parasitic organism classified into 17 major groups [1, 2]. All these acoelomate worms have bilateral symmetry; they are hermaphrodite with some exceptions and have a simple centralized nervous system and a mesodermal germ layer [3, 4]. Flatworms are characterized by a high degree of morphological diversity and reproduction modes (Table 1). The phenomenon of asexual reproduction that is uncommon in the animal kingdom occurs in all major groups of flatworms. This supports the presence of a population of totipotent stem cells called “neoblasts” in free-living worms and “germ or germinal cells” on flukes and tapeworms [4]. Several human infections caused by flatworms are considered major neglected tropical diseases (NTDs) by the World Health Organization: cysticercosis, schistosomiasis, fascioliasis, paragonimiasis, and echinococcosis [5].

Developing techniques to manipulate flatworms is a growing topic in contemporary research as judged by the number of reports published during the last decade [6]. Maintenance of parasite species under laboratory conditions has been challenging and genetic manipulation is still difficult [7]. However, since the 90s, attempts have been made to identify and characterize the regions controlling the expression of genes in several species of flatworms [8]. Due to the lack of a good expression system for heterologous genes in these organisms, several mammalian cell lines have been employed as transfection targets to identify functional promoters in flatworms [9, 10]. In this regard, the recently described genomes for several of these organisms, including the free-living planarian* Schmidtea Mediterranean* [11], and the parasites* Schistosoma mansoni*,* S. japonicum* [12, 13],* Taenia solium* [14, 15],* Echinococcus granulosus,* and* E. multilocularis* [15] represent a considerable advantage. Those genome projects allowed us to identify orthologous genes of each species and group and their functional promoters as well as to carry out* in silico* metagenomic studies. Transfection studies for each of the three groups of Platyhelminthes done so far are described in this short review.

### 1.1. Tricladida

Planarians have the capacity of regenerating complete worms from a small fragment of their bodies [16]. In 1981, Baguñà described a group of cells conferring these regenerative properties as “neoblasts” [16–18]. In order to understand the basis of tissue regeneration in these flatworms, several studies were conducted [18], which could represent a valuable contribution to human regenerative medicine [16] as well as to the establishment of stable germ cell lines useful in transfection studies [19]. However, it was not until the advent of the molecular biology and genetic tools that further investigation in this phenomenon was possible. Thus, in 1999 the Dglvs gene (*Dugesia* VASA-like) was reported as the first gene expressed in neoblasts [20] and, almost simultaneously, a successful application of RNA interference (RNAi) was reported [21]. Since then, several related neoblast genes have been described and strategies for transient transfections have been developed for Tricladida [16]. The most used method for introducing exogenous genes in the different stages of these organisms is microinjection, which is also frequently used for silencing genes such as Djpum, nanos, ß-catenin, ndk, DjFGFR1, and DjFGFR2 [16, 18, 22, 23]. This method, although highly efficient in adult flatworms, was very invasive for early developmental stages. More recently, a novel method for introducing exogenous materials into developing planarian embryos by nanosecond exposure of eggs to pulsed laser has been reported. This represents the first report of planarian embryos being genetically modified without compromising their normal development [17]. However, availability of suitable vectors for stable transfections is required to allow incorporation of exogenous genetic material into the genome of these organisms. For example, in the case of planarians three mobile elements (mariner, Hermes, and PiggyBac) have been introduced using the green fluorescent protein (EGFP: enhanced green fluorescent protein) as reporter gene, using microinjection followed by electroporation to transfect the parenchymal cells of adult flatworms [19]. Until now, the three transposons have shown good efficiency of integration into the genome in neoblast cells. PiggyBac and Hermes appear to be quite stable showing a good expression after eight months of transfection [19].

## 2. Digenean Trematodes

Trematode infections reach high prevalence in developing countries [5, 24]. The helminth infection with the largest global prevalence is schistosomiasis with 207 million cases worldwide, mainly caused by three species of blood flukes:* S. haematobium*,* S. mansoni,* and* S. japonicum*. In the case of trematodes, extensive studies on vaccines, drug development, and diagnostic methods are available [24]. Moreover, the complete genomes of* S. mansoni* and* S. japonicum* have been elucidated [12, 13]. Attempts of identifying genes and introducing heterologous genetic material have been carried out for more than a decade. New technologies have enabled success to identify, to silence, and to carry out transient transfections of several genes. Stable transfections have been achieved, allowing the approach to questions about the involvement of specific genes in disease pathogenesis or the identification of new target candidates for drug treatment [24]. Several reviews are available where the genomic history of schistosomes, including advances on transfection, is well organized [8, 10, 25–29].  Table 2 summarizes the progress in the transfection of* S. mansoni* and* S. japonicum*.

Other trematodes causing infections of high global prevalence (>40 million cases) [24], such as* Clonorchis sinensis* (liver fluke),* Opisthorchis viverrini* (liver fluke),* Paragonimus spp* (lung fluke),* Fasciolopsis buski* (intestinal fluke), and* Fasciola hepatica* (intestinal fluke), have not been successfully transfected; successful methodologies developed for* S. mansoni* could be adapted for these trematodes [27]. However, the promoter region of cathepsin 1 from* F. hepatica* has been characterized through transient transfection of mammalian Vero cells [30]. Another case is the* Paragonimus westermani* retrotransposon sequences belonging to three LTR (long terminal retrotransposons) retrotransposon families [31]. Two of these retrotransposon sequences appeared to maintain their mobile activities as suggested by the presence of mRNA transcripts [31]. The ability to integrate into the flatworm genome makes transposons and retrotransposons excellent candidates to develop stable transfections [32].

Three methods have been exploited for nucleic acid delivery into schistosomes [28]: biolistic (particle bombardment/gene gun), electroporation, and infectious retroviral vectors (Table 2). Electroporation has been considered as the most efficient method for transfection of sporocysts and schistosomules. However, biolistic has also been successfully used on miracidia and adults [33]. The choice of a delivery method depends on the organism and the life cycle stage under study. Moreover, experiences in schistosomes can also help to choose and adapt one transfection method on related organisms.

An application of transient transfection methodologies is the silencing of specific genes through RNAi, involving studies on worm viability, development, tegument physiology, egg development, signaling pathways, and drug discovery. Efficacy of RNAi can be influenced by the method of delivery: the more often used in schistosomes are soaking and electroporation [8] and the most frequently used RNAi in schistosomes is dsRNA (long double stranded), followed by siRNA (small interfering) [29]. The properties of each RNAi have been important to define their use; for example, it has been suggested that siRNA accumulates faster in certain tissues [50], whereas dsRNA is more stable to RNAse digestion [51]. In addition, dsRNA experiments are cheaper than the siRNAs counterpart [29, 51]; however, siRNAs can be more efficient inhibitors when multiple sequence oligonucleotides are used against the same target [8, 28, 29, 51, 52]. Developments of RNA silencing in schistosomes and other trematodes have accumulated during the last decade (Table 3).

Table 3 shows that although the most widely used RNAi is dsRNA gene silencing also can be efficiently achieved with siRNA [29]. The RNAi agent and the delivery method can be defined after the gene target and the stage of the parasites are selected. It is worth mentioning that initial attempts towards knocking down the expression of* S. mansoni* essential genes through* in vivo* administration of siRNA on infected hosts have produced encouraging results [53]. This strategy, that takes advantage of the low mRNA levels of the homologue gene in the host's tissues (hypoxanthine-guanine phosphoribosyl transferase: HGPRTase), is restricted in the case of other essential genes [54].

## 3. Cestodes

Among cestodes the most important infections in public health are cysticercosis and hydatosis or echinococcosis, with high global prevalence in endemic countries [5, 85]. In the case of these parasites, extensive studies on immunodiagnosis, drug and vaccine development, and so forth have been carried out [85–87]. However, transfection studies on cestodes have been scarce. An important development in the manipulation of these parasites is the isolation of germinal cells lines. For* T. crassiceps* it was possible to regenerate complete cysticerci from cellular clusters [88]; for* E. multilocularis* new metacestodes were regenerated from the germinal layer [89]; in the case of* E. granulosus,* the isolation and* in vitro* maintenance and propagation of germinal cells have been reported [90, 91]. The most significant development in the transfection of cestode parasites was achieved on* E. multilocularis* using axenic cultures of metacestodes. After some time in coculture with rat hepatocytes, the germinal cells formed a laminar layer and then clustered until the regeneration of the metacestode vesicles [92]. The first attempts of a transient transfection were done by lipofection of germinal cells with a cyanofluorescent gene as a reporter under the control of* elp* (encoding the ezrin-radixin-moesin- (ERM-) like protein), an* E. multilocularis* gene promoter [92]. Transient expression of the fluorescent protein was detected. Furthermore, these cells of* E. multilocularis* were infected with the intracellular bacterium* Listeria monocytogenes*, demonstrating a good nucleic acid carrier system [92]. The use of an attenuated, self-destructive bacteria is exciting, as it can reach the cytosol of the host cells and induce the expression of a heterologous gene under the control of the *P*_acta_ promoter [93]. This approach could be useful for other Platyhelminthes where cell lines can be isolated and maintained* in vitro*. In the case of gene silencing, an experiment in which* elp* and 14-3-3 were used as target genes, employing soaking and electroporation to deliver the siRNA, showed that the protein expression of 14-3-3 and* elp* decreases ~22% and ~72%, respectively, on day fifteen, after transfection of the protoscoleces of* E. multilocularis* [94]. Another silencing experiment was performed in the cestode of ruminants* Moniezia expansa*; the aim was to silence the transcription of actin (Me-act-1) gene using dsRNA. The reduction of actin expression was detected by immunohistochemistry and western blot techniques, in addition to severe damage in the morphology of tegument [95]. These studies demonstrated that the transfection and gene silencing techniques can be successfully used in cestodes. In fact, we have achieved successful transient transfection of* T. crassiceps* cysticerci* in vitro* by microinjection using a cytomegalovirus promoter and GFP as a reporter (submitted for publication). In addition, we are conducting assays to achieve stable transfection using PiggyBac transposon, as well as developing strategies for the introduction of dsRNA to silence target genes in* T. crassiceps*.

Thus, the genetic manipulation of cestode parasites is currently under examination with the goal of developing reliable methodologies for stable transfection and* in vitro* maintenance of cell lines.

## 4. Conclusion

The new technologies for genetic manipulation and transgenesis have been used in trematode parasites, specifically in* S. mansoni* [29], which is a starting point for other flatworms. However, the progress in helminths and especially in cestodes has been limited by the inability to produce stable cell lines, although the recent advances in* Echinococcus* are encouraging. It is important to remark that the advance in Platyhelminth parasites is still limited in comparison with the protozoan parasitic organisms, where highly reproducible transfection methods, including stable transfections [9], have been available for some time. Transposons, bacteria, viruses, and constructs with sequences that allow integration of exogenous sequences into the flatworms genome have been already used, but successful experiments have been only reported for* Schistosoma* [10, 28, 29]. It is expected that similar gains can be achieved in other flatworms. If so, molecular helminthology will be transformed from descriptive to more functional investigations. The need to develop methods for the production and* in vitro* cultivation of germ cell lines for genetic manipulation is emphasized [89].

## Figures and Tables

**Table 1:** Main characteristics of the groups where genetic transfection has been achieved^*^.

Group	Biologic interactions	Adult body	Life cycle	Example of genus
Tricladida	Mostly free-living	Nonsegmented	Simple	*Dugesia, Schmidtea *
Trematoda	Endoparasites of invertebrates and vertebrates	Nonsegmented	Complex	*Fasciola, Schistosoma *
Cestoda	Endoparasites of vertebrates	Segmented	Complex	*Taenia, Echinococcus *

**Table 2:** Transfection of heterologous genes in Schistosomes.

Species	Agent and method	Promoter	Reporter gene	Life stage transfected	Transfection type	References
*S. mansoni *	RNA and plasmid by particle bombardment	Spliced Leader	Luciferase	Adult worm	Transient	[34]
	Plasmid by particle bombardment	Hsp70	GFP	Adult worm and sporocysts	Transient	[35]
	Plasmid by particle bombardment	ER60	GFP	Female miracidia with sporocysts	Transient	[36]
	Plasmid by particle bombardment	SmCNA	GFP	Adult worm	Transient	[37]
	Plasmid by particle bombardment	ER60	GFP	Adult worm	Transient	[38]
	Plasmid by particle bombardment	Hsp70	EGFP	Miracidia	Transient	[39]
	RNA by electroporation	—	Luciferase	Schistosomula	Transient	[40]
	RNA by particle bombardment and electroporation	—	Luciferase	Sporocysts, miracidia, and adult worm	Transient	[41]
	VSVG-pseudo MMLV plasmid by cation polybrene	SL and hsp70	EGFP and Luciferase	Schistosomula	Transient	[42]
	Electroporation	SmACT1.1	Luciferase	Schistosomula	Transient	[43]
	PiggyBac by electroporation	Actin and HSP70	Luciferase	Schistosomula	Stable	[44]
	VSVG-pseudo MMLV plasmid by lipofectamine	Sma-Zinc	Luciferase	Adult worm and schistosomula	Stable	[45]
	RNA and VSVG-pseudo MMLV by electroporation	—	CY3 and luciferase	Eggs	Stable	[46]
	MLV pseudotyped plasmid by lipofectamine or polyethylenimine	MLV 5′, Pol II, *vasa-like,* Actin, Pol III U6	Luciferase and EGFP	Schistosomula, eggs, and adult worms	Stable	[47]

*S. japonicum *	Plasmid by electroporation	CMV	EGFP and luciferase	Schistosomula and adult worm	Transient	[48]
	VSVG-pseudo pantropic retrovirus plasmid by cation polybrene	LTR	hTERT	Schistosomula	Stable	[49]

SL: splice leader, hsp70: heat-shock protein 70, ER60: endoplasmic reticulum 60, SmCNA: *Schistosoma mansoni* calcineurin 1, CMV: cytomegalovirus, SmAct 1: *Schistosoma mansoni* actin 1, Sma-Zinc: *Schistosoma mansoni* Zinc finger protein, hTERT: human telomerase reverse transcriptase, VSVG: vesicular stomatitis virus glycoprotein, MMLV: Moloney murine leukemia retroviral, and LTR: retrovirus long terminal repeat.

**Table 3:** RNA silencing in trematode parasites.

Species	Rnai	Target gene	Life stage target	Silencing efficacy	References
*S. mansoni *	dsRNA	SGTP1 and GAPDH	Miracidia and sporocyst	70–80% (t); 40% (p)	[55]
	dsRNA	SmCB1	Schistosomula	10-fold (t)	[56]
	dsRNA	SmCB1 and SmCB31	Cercariae and adult worms	80% (t)	[57]
	dsRNA and siRNA	SmAP	Cercariae and adult worms	>90% (t); >70% (p)	[58]
	siRNA	SmRPNII/POH1	Schistosomula	80% (t)	[59]
	dsRNA	Cathepsin D	Schistosomula	100% (t)	[60]
	siRNA	HGPRTase	Cercariae	↓ 27% parasite load, 65% (t)	[53]
	dsRNA	SmLAP 1 and SmLAP2	Eggs	↓ 80% hatching	[61]
	dsRNA	32 genes (antioxidants, transcription factors, cellular signaling, and metabolic enzymes)	Miracidia	Mobility, growth, and viability affected	[62]
	siRNA	SmAP	Adult worms	80% (t)	[63]
	dsRNA	SmTK4	Adult worms	17–63% (p)	[64]
	dsRNA	SmAQP	Adult worms	90–95% (t)	[65]
	dsRNA	SmPAL	Adult worms	Inconsistent results	[66]
	dsRNA	SmGTP-1 and SmGTP-4	Adult worms	*In vivo*: SmGTP-1 55% (t), SmGTP-4 85% (t); *In vitro*: SmGTP-1, 70% (t), SmGTP-4, 90% (t)	[67]
	dsRNA	11 genes	Schistosomula	40–75% (t)	[68]
	dsRNA	SmCa1 and SmCa2	Miracidia	35% (p)	[69]
	sh-RNA	Luciferase	Schistosomula	47.5% (p)	[70]
	dsRNA	SmAP	Adult worms	95% (t)	[71]
	dsRNA	Sm-NPP-1	Schistosomula and adult worms	55% (t)	[72]
	siRNA	SmCD59	Schistosomula	60% (t)	[73]
	dsRNA	SmCaMK_2_, SmJNK, SmERK1, SmERK2, and SmRas	Schistosomula	SmERK1 92% (t), SmERK2 56% (t), SmRas 42% (t)	[74]
	dsRNA	SmACC-1 and SmACC-2	Schistosomula	SmACC-1 60% (t), SmACC-2 90% (t)	[75]
	siRNA	SmAP, SmNPP-5, and SmATPDase1	Schistosomula and Adult worms	SmAP 90% (t), SmNPP-5 >90% (t), SmATPDase1 80% (t)	[54]
	siRNA	Sm5HTR	Schistosomula and adult worms	Larvae: 100% (t) and ↓ 80% motility; adult male and female: 90% (t) and ↓ 60% motility, 80% (t) and ↓ 50% motility, respectively	[76]

*S. japonicum *	dsRNA	SjGCP	Adult worms	75% (t)	[77]
	siRNA	Mago Nashi	Schistosomula	66–81% (t)	[78]
	dsRNA	Prxs 1 and Prxs 2	Schistosomula and adult worms	~20% (t)	[79]
	dsRNA	(SHSP) Sjp40	Adult worms	80% (t)	[80]
	siRNA	SjAR (SiRNA1 and SiRNA2)	Schistosomula	48% (t) and 73% (t)	[81]

*S. haematobium *	siRNA and dsRNA	Luciferase and Sh-tsp-2	Eggs, schistosomula, and adult worms	>75% (p) for both	[82]

*F. hepatica *	dsRNA	FheCL and FheCB	Metacercariae	FheCL1: 80% (t)	[83]
	dsRNA	FhLAP	Young larvae	>90% (p)	[84]

SGTP: facilitated diffusion glucose transporter, GAPDH: glyceraldehyde-3-phosphato-dehydrogenase, SmCB: *Schistosoma mansoni* cathepsin B, SmAP: *Schistosoma mansoni* alkaline phosphatase, SmRPNII/POH1: *Schistosoma mansoni* proteasome subunit, HGPRTase: hypoxanthine-guanine phosphoribosyl transferase, SmLAP:* Schistosoma mansoni* leucine aminopeptidase, SmTK4: *Schistosoma mansoni* SYK kinase, SmAQP: *Schistosoma mansoni* aquaporin gene, SmPAL: *Schistosoma mansoni* peptidylglycine alpha-amidating lyase, SmGTP: *Schistosoma mansoni* glucose transporter, SmCa: *Schistosoma mansoni* calmodulin sensing, Sm-NPP-1: *Schistosoma mansoni* neuropeptide precursor 1, SmCaMK: *Schistosoma mansoni *calmodulin-binding kinase, SmJNK: *Schistosoma mansoni* C-JUN-N-terminal kinase, SmERK: *Schistosoma mansoni* extracellular signal-regulated kinase, SmRAS: small GTPase superfamily, SmACC: *Schistosoma mansoni* acetylcholine-gated chloride channels, SmHTR: *Schistosoma mansoni* serotonin-activated G protein-coupled R, SjGCP: *Schistosoma japonicum* gynecophoral canal, Prxs: peroxiredoxin, Sjp40: *Schistosoma japonicum* short heat-shock protein, SjAR: *Schistosoma japonicum *aldose reductase, FheCL and FheCB: *Fasciola hepatica* cathepsin L and B, FhLAP: *Fasciola hepatica* leucine aminopeptidase, and sh-tsp-2: transcription of tetraspanin 2. (↓): knockdown; (t): transcript; (p): protein.
